# Dramatic Repositioning of c-Myb to Different Promoters during the Cell Cycle Observed by Combining Cell Sorting with Chromatin Immunoprecipitation

**DOI:** 10.1371/journal.pone.0017362

**Published:** 2011-02-22

**Authors:** Anita M. Quintana, Ye E. Zhou, Janeth J. Pena, John P. O'Rourke, Scott A. Ness

**Affiliations:** Department of Molecular Genetics and Microbiology, University of New Mexico Health Sciences Center, Albuquerque, New Mexico, United States of America; Wellcome Trust Centre for Stem Cell Research, United Kingdom

## Abstract

The c-Myb transcription factor is a critical regulator of proliferation and stem cell differentiation, and mutated alleles of c-Myb are oncogenic, but little is known about changes in c-Myb activity during the cell cycle. To map the association of c-Myb with specific target genes during the cell cycle, we developed a novel Fix-Sort-ChIP approach, in which asynchronously growing cells were fixed with formaldehyde, stained with Hoechst 33342 and separated into different cell cycle fractions by flow sorting, then processed for chromatin immunoprecipitation (ChIP) assays. We found that c-Myb actively repositions, binding to some genes only in specific cell cycle phases. In addition, the specificity of c-Myb is dramatically different in small subpopulations of cells, for example cells in the G2/M phase of the cell cycle, than in the bulk population. The repositioning of c-Myb during the cell cycle is not due to changes in its expression and also occurs with ectopically expressed, epitope-tagged versions of c-Myb. The repositioning occurs in established cell lines, in primary human CD34+ hematopoietic progenitors and in primary human acute myeloid leukemia cells. The combination of fixation, sorting and ChIP analysis sheds new light on the dynamic nature of gene regulation during the cell cycle and provides a new type of tool for the analysis of gene regulation in small subsets of cells, such as cells in a specific phase of the cell cycle.

## Introduction

The c-Myb protein is a DNA binding transcription factor that regulates the expression of specific target genes [1]. Mutations that convert the normal c-Myb protein into an oncogenic transforming protein also change the spectrum of genes that it regulates [2], [3]. Several types of evidence suggest that the c-Myb transcription factor may be regulated during the cell cycle. For example, c-Myb interacts with Cyclin D1 [4] and with cyclin-dependent kinases CDK4 and CDK6 [5]. In addition, c-Myb has been shown to regulate the CCNB1 gene, which encodes the cell cycle regulator Cyclin B1 [6], and has been implicated in the regulation of the CCNE1 gene, which encodes Cyclin E1 [7]. The c-Myb transcription factor is responsible for the proper regulation of hematopoiesis [8] and inhibition or ablation of c-*myb* gene expression blocks hematopoietic cell differentiation *in vitro* [9] and leads to a loss of hematopoietic cells in animals [10]. Mutations in protein interaction sites in c-Myb lead to defects in hematopoietic stem cell differentiation [11], and change the specificity of c-Myb, allowing it to regulate different sets of target genes [2], [3], [12]. Thus, oncogenic mutations could alter the activity of c-Myb, a transcription factor that is normally regulated during the cell cycle.

But how would cell cycle regulation of c-Myb manifest itself? At least two mechanisms have been described for transcription factor regulation during the cell cycle. One example is E2F transcription factors, which are inhibited during the G1 phase of the cell cycle by bound Retinoblastoma tumor suppressor protein. In S phase, Cyclin D1/CDK4 phosphorylation of Retinoblastoma triggers its removal and leads to the activation of E2F target genes [13]. In contrast, receptor-activated signaling cascades lead to activation of kinases like Akt, which phosphorylate FOXO transcription factors, leading to their migration to and sequestering in the cytoplasm, preventing them from regulating the expression of genes encoding cell cycle regulators [14]. Both of these examples are ways in which transcription factors change activity or localization, but not specificity, during the cell cycle.

We set out to monitor the activity of c-Myb and determine whether it is regulated during the cell cycle. We opted to use chromatin immunoprecipitation to follow the association of c-Myb with different gene promoters in cultures of human cells that were progressing normally through the cell cycle. This approach allowed us to fix the proteins with formaldehyde while the cells were still in the culture dish, locking in the results before the cells were manipulated in any way. Then the cells were harvested, sorted into cell cycle stages and used for chromatin immunoprecipitation experiments. The results show that c-Myb undergoes dramatic and dynamic repositioning onto different gene promoters during the cell cycle, suggesting that complex mechanisms regulate its specificity and activity in a time-dependent manner, and illustrating a novel mechanism for transcription factor regulation during the cell cycle.

## Results

### Hydroxyurea and nocodazole cause dramatic changes in c-Myb expression and activity

We were faced with a dilemma when we set out to measure the activities of c-Myb during different phases of the cell cycle, since conventional methods of measuring transcription factor activity are poorly suited to such studies. For example, reporter gene assays have been used to measure c-Myb activity, but rely on the production of reporter enzymes, which must accumulate and could have long half-lives. Similarly, target gene mRNAs could be present long after c-Myb did its work. We settled on the approach of using chromatin immunoprecipitation (ChIP) assays to detect promoters c-Myb was associated with during specific phases of the cell cycle. We focused on two cell cycle-dependent target genes for our study. The CCNB1 gene, which encodes Cyclin B1, was recently shown to be a bona fide c-Myb target gene in hematopoietic cells [6]. The CCNE1 gene, encoding Cyclin E1, has been implicated as a c-Myb target through co-localization and knockdown studies in several cell types [7]. Both of the cyclin genes are expected to be regulated in a cell cycle-specific fashion, so should be excellent models for following changes in c-Myb activity. Both genes have been confirmed as bona fide c-Myb targets through genome-wide promoter tiling array experiments (Quintana AM, Liu F, O'Rourke JP and Ness SA, manuscript submitted), which have been deposited in the NCBI GEO database (accession number: GSE18706).

**Figure 1:**
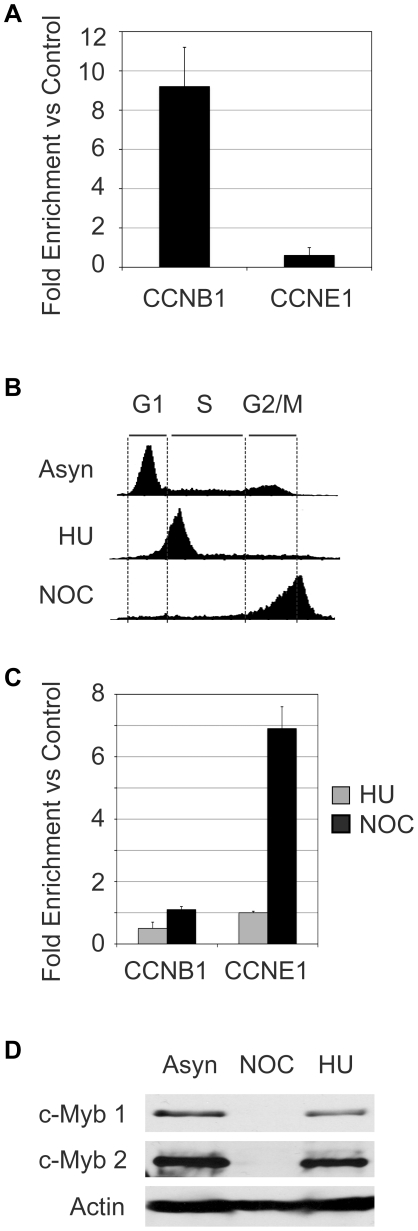
Hydroxyurea and nocodazole affect c-Myb levels and activity. (A) Conventional ChIP assay with Jurkat cells. Asynchronously growing Jurkat cells were fixed and subjected to ChIP analysis to determine whether c-Myb was associated with the *CCNB1* and *CCNE1* promoters. The results are plotted as fold enrichment compared to control IgG as measured using Quantitative real-time PCR (QPCR). Error bars show the standard deviation of triplicate QPCR assays. The results are representative of several independent experiments. (B) Effects of hydroxyurea (HU) and nocodazole (NOC). Jurkat cells were treated 16–18 hr with the cell cycle blockers HU or NOC to arrest the cells in S phase or G2/M, respectively, then samples were stained with Hoechst 33342 and the cell cycle profiles were compared to untreated, asynchronously (Asyn) growing cells by flow cytometry. The histograms show cell number vs DNA content, and the G1, S and G2/M portions of the diagrams are labeled. (C) ChIP assay with HU and NOC treated cells. Jurkat cells treated 16–18 hr with HU or NOC were harvested, fixed and processed for ChIP assays, using anti-Myb antibodies. The association of c-Myb with the *CCNB1* or *CCNE1* promoters was assessed by QPCR, as described above. Results are plotted as fold enrichment compared to control IgG and error bars show standard deviation of triplicate QPCR assays. These results are representative of several independent experiments. (D) Expression of c-Myb protein. Total cell extracts from Jurkat cells growing asynchronously (Asyn) or treated with NOC or HU were analyzed by Western blot for expression of c-Myb protein. The blot was treated with one anti-Myb antibody, then stripped and re-probed with a different anti-c-Myb antibody and finally stripped and re-probed for beta-actin as a protein loading control. These results are representative of several independent experiments. Note: All of the experiments in this figure were performed at least three times each. The results shown are from a single experiment but are representative of all the trials.

Initial ChIP assays using asynchronously growing Jurkat T-cells showed c-Myb protein associated with the promoter of the CCNB1 genes, but not with the CCNE1 gene promoter (Figure 1A). To follow changes during the cell cycle, we treated the cells with hydroxyurea (HU) or nocodazole (NOC) for 18 hr to arrest the cells in early S phase or G2/M, respectively (Figure 1B). However, when ChIP assays were performed on the drug-treated cells, c-Myb could not be detected at either the CCNB1 or CCNE1 promoters in the hydroxyurea treated cells (Figure 1C, gray bars), although c-Myb was associated with the CCNE1 promoter in the nocodazole-treated cells (Figure 1C, black bars). Thus, the cells treated with hydroxyurea or nocodazole gave results in the ChIP assay that were completely different than the asynchronously growing cells, suggesting that c-Myb protein activity could be cell cycle regulated.

Next we analyzed these cell populations for c-Myb protein expression by Western blot. We found that c-Myb protein levels were comparable in the asynchronously growing and hydroxyurea-treated cells, but were dramatically reduced in the cells treated with nocodazole (Figure 1D). We obtained this result in multiple experiments and using two different anti-c-Myb antibodies, suggesting that nocodazole treatment leads to a dramatic decrease in c-Myb protein levels in Jurkat T cells. Note: Although the Western blot shown in Figure 1D gives the impression that c-Myb protein is absent in the nocodazole-treated cells, c-Myb protein is detectable in longer exposures (not shown). The residual protein is presumably responsible for the positive ChIP results in the nocodazole-treated cells (Figure 1C). The stability and degradation of c-Myb protein has been reported to be controlled by multiple different regulatory pathways [15]–[18]. Our results using hyrdroxyurea- and nocodazole-treated cells suggest that these pathways may be affected by the drug treatments or the cell cycle. This makes the use of hydroxyurea and nocodazole problematic for the study of c-Myb during the cell cycle, which prompted us to develop a different approach.

### The levels of c-Myb associated with chromatin remain constant during the cell cycle

**Figure 2:**
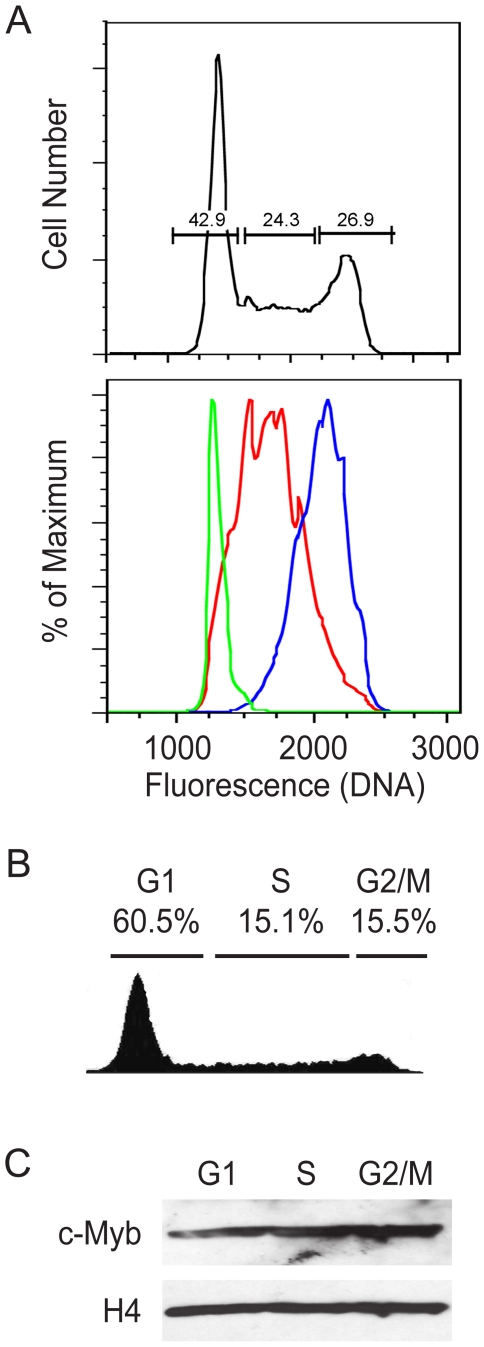
Chromatin-associated levels of c-Myb remain constant during the cell cycle. (A) Cell cycle sorting of formaldehyde fixed cells. Asynchronously growing Jurkat cells were fixed with formaldehyde, stained with Hoechst 33342 then subjected to cell sorting, using a sorter equipped with a UV laser. The top panel shows the original cell cycle distribution and the gates that were used to collect the three cell cycle fractions. The lower panel shows the results of re-analyzing the cells collected in each sorted fraction. (B) Jurkat cell cycle analysis. Cell cycle histogram of a representative culture of Jurkat T cells progressing through the cell cycle. The cells were fixed with formaldehyde, stained with Hoechst 33342 and analyzed by flow cytometry. The fraction of cells in each cell cycle phase is indicated at the top. (C) Expression of c-Myb. Jurkat cells were fixed, stained with Hoechst 33342 and sorted into G1, S or G2/M fractions. Total chromatin was prepared from equal numbers of cells in each cell cycle fraction, then the cross-links were reversed and the samples were analyzed by Western blot (D) for expression of c-Myb protein. The blot was stripped and re-probed for histone H4 as a loading control. Note: The experiments in this figure were performed at least twice. The results shown are from a single experiment but are representative of all the trials.

Our studies with the drug-treated cells showed dramatic changes in c-Myb protein levels in the cells that were arrested in G1, S and G2/M (Figure 1). However, those changes were in conflict with our ChIP assay results, which showed that c-Myb must be bound to at least some promoters in the G2/M phase of the cell cycle. These conflicting results prompted us to develop a different type of cell cycle assay that did not rely on drug treatments. First, we tested whether asynchronously growing cells could be fixed with formaldehyde, as for a ChIP assay, then stained with Hoechst 33342 DNA content dye and subjected to flow cytometric cell sorting. As shown in Figure 2A, the fixed Jurkat cells gave a typical cell cycle profile when analyzed for DNA content. We sorted the cells into G1, S and G2/M fractions, then re-analyzed the sorted cells (Figure 2A, lower panel), which showed that, although there was some overlap in the DNA content of the sorted cells, the fractions were greatly enriched for cells in the expected cell cycle stages.

Since c-Myb is a DNA-binding transcription factor, the active fraction of the protein is presumed to be associated with chromatin. Therefore, we determined the c-Myb protein levels that were associated with chromatin during the different cell cycle stages. We fixed and sorted an asynchronously growing culture of Jurkat cells (Figure 2B), harvested the chromatin from equal numbers of fixed cells from the G1, S or G2/M cell cycle fractions, then de-crosslinked the samples and analyzed the recovered proteins by Western blot using anti-c-Myb antibodies. As shown in Figure 2C, all three cell cycle fractions contained the same amount of Histone H4 (used here as a loading control) and very similar amounts of c-Myb protein, suggesting that c-Myb protein expression, localization and association with chromatin and DNA remained constant throughout the cell cycle. This is an important result, which contrasts sharply with the results we obtained with the drug-treated cells. Our analysis using cells that were rapidly fixed and sorted based on DNA content shows that the levels of c-Myb associated with chromatin remain constant, suggesting that c-Myb could be associated with and regulate target genes throughout the cell cycle.

### Association of c-Myb with some target genes is cell cycle stage specific

The results using the fixed and sorted cells (Figure 2) suggested that we could extend that approach to analyze the association of c-Myb with target genes in ChIP assays. With that in mind, we developed the Fix-Sort-ChIP approach, in which asynchronously growing cells were fixed with formaldehyde, stained and sorted into cell cycle fractions as described in the previous section, then the chromatin was recovered from equal numbers of sorted cells in each cell cycle stage and processed for ChIP assays.

**Figure 3:**
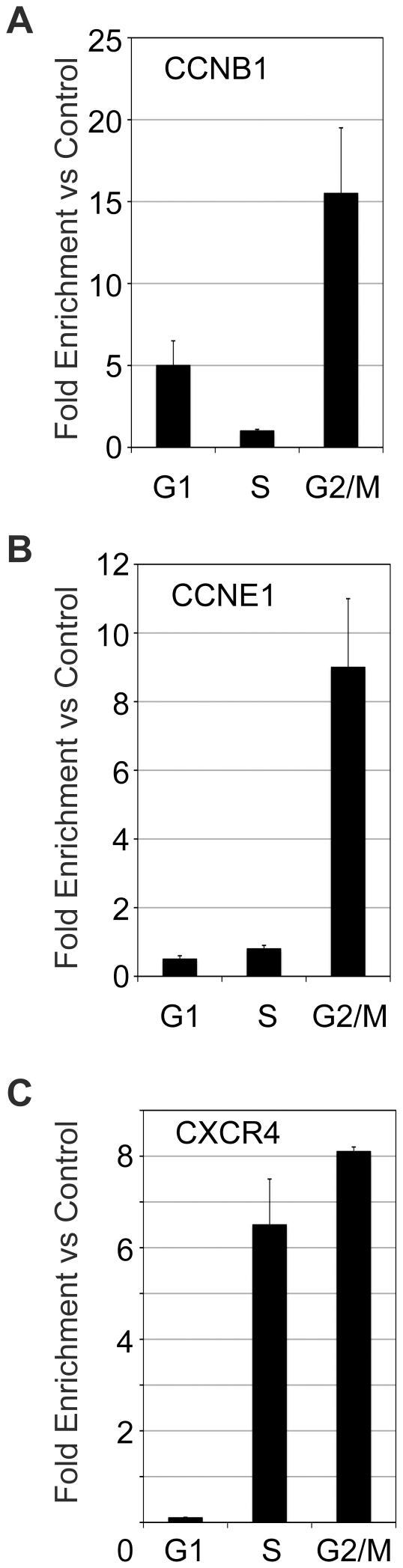
Redistribution of c-Myb during the cell cycle. Jurkat cells were fixed with formaldehyde, stained with Hoechst 33342, fractionated into cell cycle fractions by fluorescence-activated cell sorting, then chromatin from equal numbers of cells in different cell cycle fractions was analyzed by ChIP using anti-Myb antibodies. The association of c-Myb with known target genes (A) *CCNB1*, (B) *CCNE1* or (C) *CXCR4* is shown. Results are expressed as fold enrichment relative to control IgG, and normalized to a control site in the *GAPDH* promoter, to which c-Myb does not bind. Error bars show standard deviation of triplicate QPCR reactions. These results are representative of several independent experiments. Note: The experiments in this figure were performed more than five times and two different anti-Myb antibodies were used for the ChIP assays, which gave similar results. The results shown are from a single experiment but are representative of all the trials.

Using the Fix-Sort-ChIP approach, we tested the association of c-Myb with the CCNB1 and CCNE1 gene promoters. The results from one experiment, which are representative of many similar ones, are shown in Figure 3. As shown in Figure 3A, c-Myb was associated with the CCNB1 promoter in both G1 and G2/M, but not in S phase cells. This is consistent with reports that c-Myb regulates the CCNB1 gene in G2/M phase cells [6], but contrasts markedly with the results obtained with the drug treated cells (Figure 1C), which did not detect c-Myb at the CCNB1 promoter in the cell cycle-arrested cells. Thus, the Fix-Sort-ChIP approach appears to give better and more reliable cell cycle results than the drug treatment experiments.

Although equal amounts of c-Myb protein are bound to chromatin in all of the cell cycle phases (Figure 2D), the results in Figure 3B show that c-Myb only associates specifically with the CCNE1 promoter in the G2/M phase of the cell cycle. This association was not detected in the asynchronous cells (Figure 1A), perhaps because the G2/M phase cells made up such a small percentage of the total population. This is a striking result, suggesting that c-Myb activity changes during the cell cycle, leading it to associate with specific genes only in the G2/M phase. It also shows that c-Myb has different activities in small subpopulations of cells, which could be overlooked in a mixed population of cells. In this case, the ChIP assays failed to detect c-Myb at the CCNE1 target gene in asynchronous cells, but did so when cell sorting revealed the differences in the G2/M subpopulation. Interestingly, c-Myb was not associated with either of the cyclin genes in the S phase cells.

The cyclin genes are intimately related to control of the cell cycle, so they could represent special cases of genes that are extraordinarily regulated during the cell cycle. Therefore, we also analyzed the association of c-Myb with the CXCR4 gene promoter. The CXCR4 gene encodes the receptor for the chemokine SDF-1 and was identified as a c-Myb target gene in microarray studies [2], [3], [19], but is not known to be cell cycle regulated. Using the Fix-Sort-ChIP approach, we found c-Myb associated with the CXCR4 gene promoter in both S phase and G2/M phase cells (Figure 3C). This is an important result since it demonstrates that there is nothing preventing c-Myb from being detected at target gene promoters in S phase cells. It also shows that CXCR4 may be a cell cycle regulated gene, since c-Myb only binds the promoter in the cells that are progressing through the cell cycle, either in S phase or G2/M.

The results shown in Figure 3 are representative of numerous independent experiments, and have been reproduced using two different anti-c-Myb antibodies that recognize different parts of the protein. Therefore, we do not think the cell cycle phase-specific results are likely to be due to epitope masking or other antibody-related effects. Taken together, these results suggest that c-Myb redistributes to different target gene promoters in different phases of the cell cycle, suggesting that its specificity is regulated in a dynamic and cell cycle dependent manner.

### Association of c-Myb with target genes correlates with their expression

**Figure 4:**
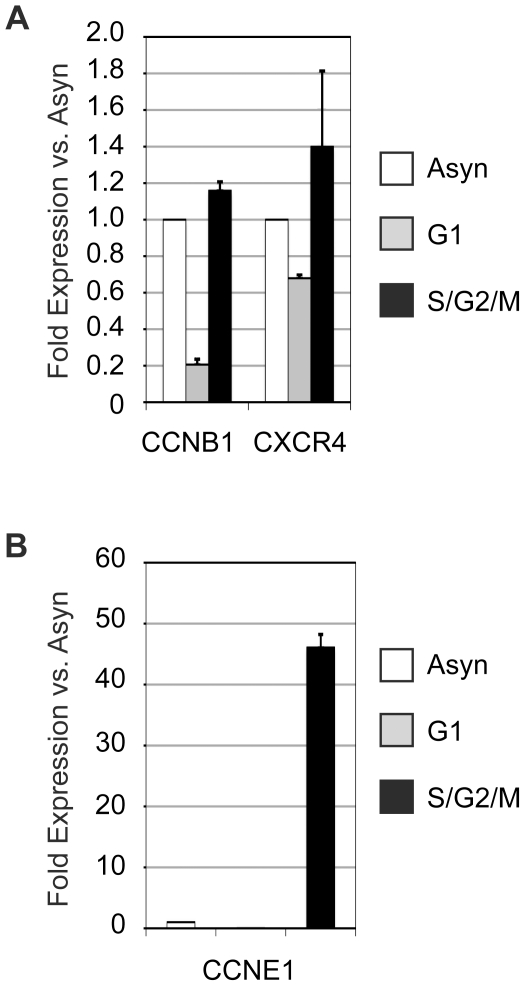
Expression of cyclin target genes correlates with c-Myb binding. Asynchronously growing Jurkat cells were collected, stained and sorted into G1 or combined S/G2/M phase fractions, then RNA was isolated and analyzed for the expression of the (A) CCNB1 or CXCR4 or (B) CCNE1 gene mRNAs using quantitative real-time PCR. The results were normalized to housekeeping genes GAPDH and PPIA and to the expression levels detected in the asynchronously growing cells, which were arbitrarily set to 1.0. Note: The experiments in this figure were performed at least twice. The results shown are from a single experiment but are representative of all the trials.

The results of the ChIP assays shown above suggest that c-Myb is only associated with some target genes at specific times in the cell cycle. We next tested whether the expression of these target genes correlated with the binding of c-Myb to their promoters. However, because isolating RNA from formaldehyde-fixed cells was not possible, we used unfixed cells for the gene expression assays, and performed the sorting as quickly as possible to preserve the RNA, so we could only harvest enough cells to produce G1 and combined S/G2/M populations. Briefly, asynchronously growing Jurkat T cells were stained and sorted into G1 or combined S/G2/M fractions as described above, but without the formaldehyde fixation that interfered with recovery of RNA. The sorted cells were immediately processed to purify the RNA, which was analyzed by quantitative real-time PCR. As shown in Figure 4A, CCNB1 mRNA was detected at similar levels in the asynchronously growing cells and in the S/G2/M fraction, but was about 5-fold lower in the G1 phase cells, a result which correlates nicely with the ChIP data shown in Figure 3A, where association of c-Myb with the CCNB1 promoter was several fold higher in the G2/M phase cells compared to the G1 phase cells. The results with the CCNE1 mRNA were also consistent with the results of the ChIP experiments. The S/G2/M phase cells contained more than 40-fold more CCNE1 mRNA than the asynchronous population or the G1 phase cells (Figure 4B), suggesting that the CCNE1 gene is only expressed in the S/G2/M phase cells, when c-Myb is bound to its promoter. In contrast, the CXCR4 mRNA was found at similar levels in asynchronously growing cells and in the G1 and S/G2/M fractions (Figure 4A). This could be because expression of the CXCR4 mRNA is independent of c-Myb, because the CXCR4 gene is regulated by c-Myb in some cell cycle phases and by other, unknown transcription factors in G1 phase cells (where c-Myb is not bound to the CXCR4 gene promoter) or because the CXCR4 mRNA is stable enough that its steady-state levels do not change significantly during the cell cycle. This is supported by flow cytometric analysis using anti-CXCR4 antibodies showing that CXCR4 cell surface expression remains constant during the cell cycle in Jurkat cells (data not shown). Taken together, our results suggest that the cell cycle-dependent expression of the CCNB1 and CCNE1 genes correlate with the binding of c-Myb to their promoters, but the results with the CXCR4 gene remain inconclusive.

### Ectopically expressed c-Myb is also retargeted during the cell cycle

**Figure 5:**
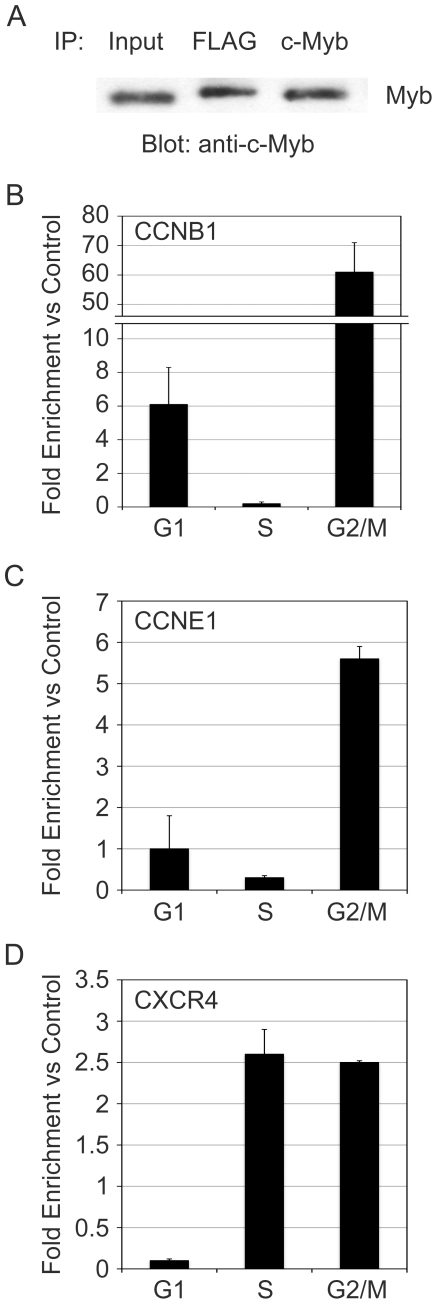
FLAG-tagged c-Myb is also repositioned during the cell cycle. (A) Expression of FLAG epitope-tagged c-Myb. Whole cell extract (Input) from Jurkat cells stably expressing FLAG-tagged c-Myb were subjected to immunoprecipitation using anti-FLAG or anti-Myb antibodies, then the recovered proteins were analyzed by Western blotting using anti-Myb antibodies. Stably-transduced Jurkat cells expressing FLAG epitope-tagged c-Myb were fixed and sorted and equal numbers of cells from the asynchronous (Asyn) population or the G1, S or G2/M cell cycle fractions were subjected to ChIP assays as described in Figure 4, using anti-FLAG or IgG (control) antibodies. Quantitative real-time PCR assays were used to measure the enrichment of the (B) *CCNB1* and (C) *CCNE1* or (D) *CXCR4* gene promoters. Error bars show standard deviation from triplicate QPCR reactions, and results are typical of multiple experiments. Note: The experiments in this figure were performed at least twice. The results shown are from a single experiment but are representative of all the trials.

The control of c-Myb protein expression is complex and involves many types of regulation, including controls of c-*myb* gene expression and estrogen regulated RNA elongation [20], [21], complex patterns of alternative RNA splicing [22] and several regulatory microRNAs that interact with the 3′-untranslated region (3′-UTR) of the c-*myb* mRNA [23]–[27]. It was possible, for example, that our ChIP assays were detecting different variants of c-Myb, produced through alternative RNA splicing, that bound to different promoters. To address these potential mechanisms, we generated an N-terminal FLAG epitope-tagged derivative of c-Myb, expressed from a lentiviral vector, which we used to transduce the Jurkat T-cells so that it was stably expressed. The lentivirus vector lacked the normal c-*myb* 3′-UTR and the microRNA binding sites, and had its own promoter that was not subject to the complicated controls regulating expression of the endogenous c-*myb* gene. It also expressed a c-Myb cDNA, so could not generate the many alternative splicing isoforms that are encoded by the normal c-*myb* gene. As shown in Figure 5A, immunoprecipitation with anti-FLAG or anti-Myb antibodies, followed by Western blot analysis with anti-Myb antibodies, shows that the FLAG-tagged derivative was stably expressed and ran slightly higher on the gels than the endogenous c-Myb protein, due to the FLAG epitope tag.

Using the cells that stably expressed the FLAG-tagged c-Myb, we repeated the Fix-Sort-ChIP experiments described above, but used anti-FLAG antibodies in the ChIP assays. As shown in Figure 5B, the FLAG-tagged c-Myb was associated with the CCNB1 promoter in G1 and more so in G2/M phase cells, but not in S phase cells. This is very similar to the results obtained with the endogenous c-Myb protein, using anti-Myb antibodies in the ChIP assay (Figure 3A). The FLAG-tagged c-Myb also mimicked endogenous c-Myb with CCNE1, binding only in G2/M phase cells (Figure 5C), and with CXCR4, binding in both S phase and G2/M, but not in G1 phase cells (Figure 5D).

The results with the ectopically expressed, FLAG-tagged c-Myb are important because they provide evidence of what mechanisms may be responsible for the retargeting of c-Myb during the cell cycle. The complex mechanisms that control c-*myb* gene expression must not play a role, and the results cannot be due to different activities of variant isoforms produced through alternative RNA splicing, since the lentivirus vector expresses only a single, full-length cDNA of c-Myb and is not subject to the controls that regulate c-*myb* gene expression. Furthermore, the microRNAs that control the stability and translation of c-*myb* mRNA do not affect the lentivirus-expressed form, which lacks the normal c-*myb* 3′-UTR. Since these experiments used the anti-FLAG antibodies, they also rule out the possibility that the results with the anti-Myb antibodies were due to epitope masking or some other antibody-specific effect. We conclude that this experiment rules out all but post-translational mechanisms, such as changes in post-translational modifications or protein-protein interactions in regulating which genes c-Myb associates with during different phases of the cell cycle.

### Cell cycle dependent targeting of c-Myb also occurs in primary cells and leukemias

All of the experiments we have reported so far were performed with Jurkat T-cells, a long-established, immortalized cell line that expresses relatively high levels of c-Myb [5], [22]. Although Jurkat cells are a good model for c-Myb in leukemias, which often have rearranged or over-expressed c-*myb* genes [26], there was a possibility that the cell cycle-dependent results were due to some Jurkat cell-specific regulatory process. Therefore, we analyzed two primary cell models with unique c-Myb target genes to rule out that the cell cycle dependent changes we had observed were somehow specific to T-cells or Jurkat cells.

**Figure 6:**
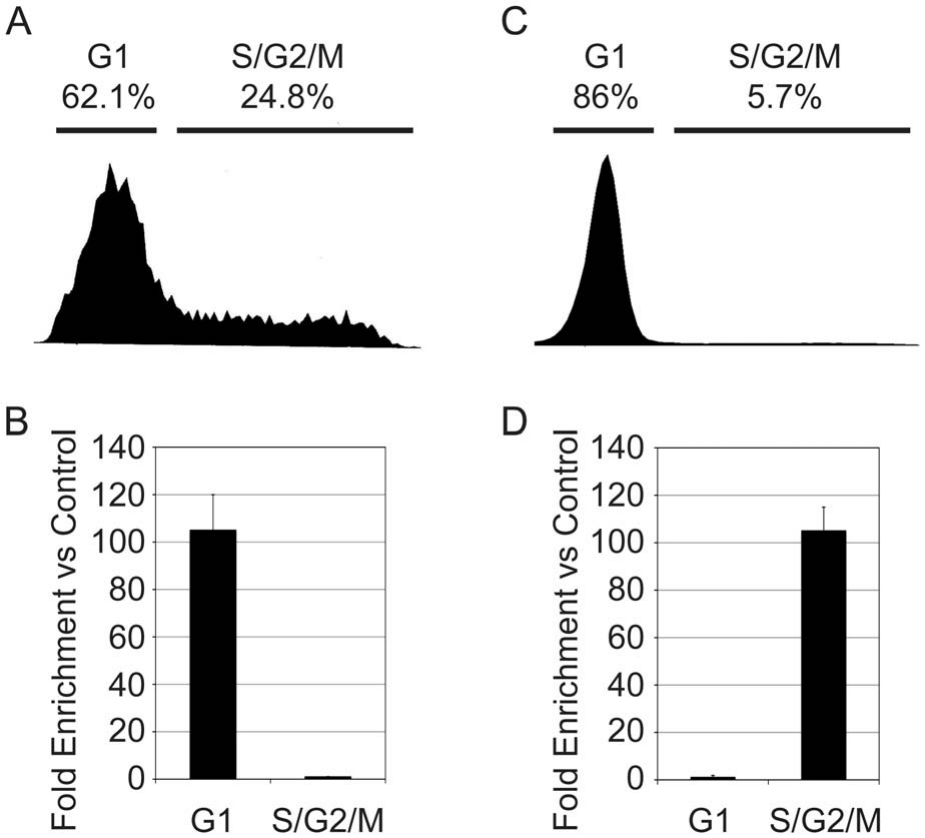
Cell cycle specific targeting of c-Myb in primary cells and leukemias. (A) Cell cycle histograms of primary CD34+ cells that were fixed and sorted into G1 or S/G2/M cell cycle fractions. The percentage of cells in each fraction is indicated at top. (B) Chromatin was prepared from equal numbers of CD34+ cells that were fixed and sorted into the G1 or S/G2/M fractions and used for ChIP assays as described in Figure 3, using anti-Myb antibodies. QPCR assays were used to measure enrichment of the *KIT* gene promoter. Results are relative to enrichment of a control region of the GAPDH gene to which c-Myb does not bind. Error bars show standard deviation of triplicate QPCR assays. (C) Cell cycle histogram of cryopreserved, primary human AML cells that were thawed and immediately fixed with formaldehyde, stained with Hoechst 33342 and sorted into G1 or S/G2/M fractions. (D) Equal numbers of sorted AML cells from each fraction were used to prepare chromatin and perform ChIP assays using anti-Myb antibodies. QPCR was used to measure the enrichment for the *CXCR4* gene promoter, as described in Figure 3. Results are relative to enrichment of a control region of the GAPDH gene to which c-Myb does not bind. Error Bars show standard deviation of triplicate QPCR reactions. Note: The experiments in this figure were performed at least twice, using cells from two different donors or patients, and all gave similar results. The results shown are from a single experiment but are representative of all the trials.

First, we used the Fix-Sort-ChIP approach to assess the association of c-Myb with the promoter of the KIT gene in primary human CD34+ hematopoietic progenitors. Several reports have implicated c-Myb as a bona fide regulator of the KIT gene, which encodes the c-Kit cell surface receptor of Stem Cell Factor or SCF [28]–[30]. Hematopoietic progenitor cells express c-Kit on their cell surface and can be stimulated to proliferate by culturing them in the presence of the c-Kit ligand SCF [31]. The primary CD34+ hematopoietic progenitor cells were fixed then stained with Hoechst 33342 and sorted into G1 or a combined S/G2/M fraction. As shown in Figure 6A, approximately 62% of the cells were in the G1 phase, and the rest were in the S/G2/M fraction. When equal numbers of cells were analyzed by ChIP assay, c-Myb was only found associated with the KIT gene promoter in the G1 cells, and not in the S/G2/M cells (Figure 6B). This is a striking result showing that c-Myb is specific for some genes, in this case KIT, only in specific phases of the cell cycle, even in primary hematopoietic progenitor cells.

We also used the Fix-Sort-ChIP approach to analyze c-Myb association with target genes in primary human acute myeloid leukemia (AML) samples. Here, we again assayed the association of c-Myb with the CXCR4 gene, whose expression has been implicated in poor prognosis in AML [32], [33]. In this case, a vial of cryopreserved bone marrow cells from an adult AML patient was thawed and immediately fixed with formaldehyde, then the cells were stained with Hoechst 33342, sorted into the G1 or S/G2/M phase samples, and then equal numbers of cells from the two fractions were subjected to ChIP analysis. As shown in Figure 6C, nearly all the AML cells were in the G1 (or G0) phase of the cell cycle. However, c-Myb associated with the CXCR4 gene promoter exclusively in the cells that were in the S and/or G2/M phase (Figure 6D). This echoes the result obtained with Jurkat cells, in which c-Myb associated with the CXCR4 gene promoter in both S and G2/M phase cells, but not G1 phase cells. Again, this highlights the fact that samples can contain small subpopulations of cells, in this case the 6% or so of AML cells in the S/G2/M phase of the cell cycle, which have dramatically different properties, or in which c-Myb is associated with totally different sets of target genes. Based on these results, we conclude that c-Myb is bound to specific genes in distinct phases of the cell cycle, suggesting that its ability to bind stably to promoters depends on cell cycle-specific changes in protein-protein interactions.

## Discussion

We have described the novel combination of cell fixation, cell sorting and chromatin immunoprecipitation (Fix-Sort-ChIP assay) as a means of following the localization of transcription factors, in this case c-Myb, during different phases of the cell cycle. The Fix-Sort-ChIP approach expands the usefulness of ChIP assays for the analysis of subsets of cells within a population. The use of fixed cells for cell sorting preserves the integrity of the chromatin complexes during the cell sorting, which can take hours if small subpopulations of cells are being purified. We also showed that FLAG epitope-tagged c-Myb repositioned in the same manner as the endogenously expressed protein. These results ruled out a number of regulatory mechanisms that could be affecting c-Myb specificity during the cell cycle, and opened the door for future structure-function studies aimed at defining the elements and domains within the c-Myb protein that control its activity and specificity during the cell cycle.

Perhaps the most striking result from our Fix-Sort-ChIP assays was the realization that c-Myb activity and specificity changed so dramatically in subpopulations of cells in different phases of the cell cycle. For example, in Jurkat cells, c-Myb associated with the promoter of the Cyclin E1 gene (CCNE1) only in G2/M phase cells. However, Western blots showed that the protein was expressed at equal levels in the different cell cycle fractions. Thus, c-Myb appeared to be targeted to specific promoters in distinct phases of the cell cycle. Our results cannot distinguish whether the access of c-Myb to the CCNE1 promoter is blocked in G1 and S phase cells, or whether some change at the CCNE1 promoter in G2/M phase cells allowed the c-Myb protein to form a stable complex. However, the association of c-Myb with the CCNE1 promoter must be dynamic: the complex must be stabilized during G2/M and de-stabilized in other parts of the cell cycle. Similarly dramatic differences were observed in CD34+ hematopoietic progenitors and in primary human AML samples, where c-Myb associated only with the KIT gene in G1 phase cells and only with the CXCR4 gene in S/G2M phase cells, respectively. Thus, the dramatic cell cycle-dependent repositioning of c-Myb is not limited to Jurkat cells, but also occurs in primary cells and leukemias.

The DNA binding domain of c-Myb is highly conserved amongst vertebrates and even insects and plants [34], [35], and is presumed to control the specificity of c-Myb by recognizing and forming stable complexes at the appropriate promoters. However, the c-Myb transcription factor recognizes a relatively simple sequence motif, (C/T)AAC(G/T)G [1], [36] which is expected to occur on average about once per kilobase, or approximately three million times in the human genome. Clearly, other determinants such as combinatorial protein-protein interactions must play important roles in regulating the specificities of transcription factors like c-Myb [37]. But protein interactions are regulated dynamically, through post-translational modifications and other mechanisms, suggesting that the specificity of DNA binding transcription factors like c-Myb, and the set of target genes that it binds to, could change rapidly in a time-dependent manner.

The cell cycle regulation of c-Myb is reminiscent of NF-Y, the ubiquitously expressed transcription factor that binds CCAAT-box promoter elements [38], [39]. NF-Y, which has histone-like subunits, displaces nucleosomes from multimeric CCAAT-boxes at the promoters it regulates and stimulates histone acetylation and gene activation [40], [41]. The c-Myb transcription factor is also involved in chromatin remodeling, via its Myb/SANT domain, which is related to components of chromatin remodeling complexes [42], [43]. The c-Myb Myb/SANT domain binds to histone tails and promotes their acetylation, stimulating the remodeling of chromatin and gene activation [44]. Thus, NF-Y and c-Myb may share the ability to initiate the remodeling of chromatin and the activation of cell cycle regulated genes.

A major unanswered question concerns how c-Myb and NF-Y become targeted to the appropriate promoters at the correct times in the cell cycle. NF-Y regulates different classes of promoters at different times in the cell cycle, but it is thought to activate constitutively: the actual cell cycle-dependent regulation occurs through other factors, like E2F, that repress or activate the promoters to which NF-Y is bound at specific times in the cell cycle [45]. In contrast, the regulation and specificity of c-Myb appears to be regulated during the cell cycle and may be linked to its interactions with Cyclin D1/CDK complexes, since phosphorylation at specific residues could change the affinity of c-Myb for promoters by altering its ability to interact in a combinatorial fashion with other proteins [37]. Several types of evidence have shown that the specificity of c-Myb can be affected by mutations [2], [3], [12] and that c-Myb regulates completely different sets of target genes in different cell types [3], [19], suggesting that its specificity is controlled by interactions with other proteins. Our new results suggest that these interactions and the activities of c-Myb are not only tissue-specific, but also change dynamically during the cell cycle.

A final question is whether the changing specificity of c-Myb is linked to its potential oncogenic activity. We have shown that the mutations that render c-Myb oncogenic also completely change its specificity, leading to the activation of different sets of target genes [2], [3]. Our discovery of cell cycle specific changes in c-Myb activity raise the possibility that oncogenic versions of c-Myb may transform cells by regulating some target genes in the incorrect stage of the cell cycle, which could stimulate cell cycle progression or inactivate cell cycle checkpoints. There is evidence that oncogenic versions of c-Myb push transformed cells through the cell cycle, even in the absence of mitogenic growth factor signals [46]. There is also a difference in the interactions between Cyclin D1 and normal or oncogenic versions of c-Myb [4]. Thus, oncogenic versions of c-Myb may have altered cell cycle-dependent activities, which could be the key to their transforming activities. Although our results demonstrate that c-Myb must relocate to different genes in different parts of the cell cycle, we have only analyzed a few target genes. It will be necessary to extend the Fix-Sort-ChIP approach to genome-wide assays to determine how general this relocating is and to identify the subsets of target genes that c-Myb regulates in different stages of the cell cycle.

## Materials and Methods

### Cells and Culture Conditions

Human Jurkat T-cells (#TIB152, ATCC Manassas, VA) were cultured at 37°/5% CO2 in RPMI + Glutamax medium (Invitrogen, Carlsbad, CA) supplemented with 10% (v/v) fetal bovine serum (Invitrogen, Carlsbad, CA). Cytokine-mobilized CD34+ cells (Fred Hutchison Cancer Research Center Large-Scale Cell Processing Core) were cultured in IMDM media (Invitrogen, Carlsbad, CA) supplemented with BITS serum substitute, IL-3 (20 ng/ml), IL-6 (20 ng/ml), Stem Cell Factor (100 ng/ml), and FLT-3 ligand (100 ng/ml) (all from Stem Cell Technology, Vancouver, Canada).

### Cell cycle Analyses

Cells were stained with 10 µg/ml Hoechst 33342 (Invitrogen, Carlsbad CA) as described [47]. Alternatively, cells were seeded at 5×10^5^/ml and treated with 1 µg/ml nocodazole (Sigma, St Louis MO) or 1 µM hydroxyurea (Sigma, St Louis MO) for 16–18 hr. Western blot assays to measure Myb protein levels and quantitative real time PCR assays for RNA levels were performed as described previously [3], [22]. RNA was isolated from equal cell numbers with RNeasy Kit (Qiagen, Valencia CA) according to manufacturer's protocol and reverse transcribed (3 ug) into cDNA template with Reverse Transcriptase III (Invitrogen, Carlsbad CA). Taqman (Applied Biosystems, Foster City, CA) real time PCR was used (except for *CCNE1*, SYBR green was used) to analyze the expression of c-*myb*, *CCNB1*, *CCND1*, and *PPIA*. For protein expression, formaldehyde-fixed proteins were extracted and purified by hydroxyapatite chromatography [48]. Briefly, cells were lysed in lysis buffer (5 M Urea, 2 M guanidine hydrochloride, 200 mM potassium phosphate buffer, and 2 M NaCl supplemented with 1 µM chymostatin, leupeptin, antipain, pepstatin-A, 1 mM each phenylmethylsulfonyl fluoride and benzamidine), incubated on pre-equilibrated hydroxyapatite beads (Biorad, Hercules, CA), de-crosslinked at 65° for 5 hours, and precipitated with chloroform: methanol (1∶4) before analysis by Western blot. Cell cycle data was analyzed using FloJo software v 9.2.

### Chromatin Immunoprecipitation (ChIP)

ChIP assays were performed by slightly modifying standard methods [49]. Briefly, approximately 1×10^6^ cells were resuspended in 500 µl of cell lysis buffer (20 mM Tris-HCl pH 8.0, 85 mM KCl, 0.5% NP-40 plus protease inhibitors mix: 1 mM each of chymostatin, leupeptin, antipain and Pepstatin A and 1 mM each of Phenylmethanesulfonyl fluoride and benzamidine, all from Sigma-Aldrich, St. Louis MO), incubated on ice for 10 min then lysed by passing up and down 2x through a 26 G needle. The nuclei were recovered by centrifugation for 10 min at 14,000× g and resuspended in 100 µl of 50 mM Tris-HCl pH 8.0 containing 5 mM CaCl2 plus protease inhibitors. The chromatin was sheared by adding 6 U of micrococcal nuclease (USB, Cleveland OH) for 10 min at 37°C, followed by adding EDTA to 10 mM and SDS to 1% and by heating to 65°C for 5 min to stop the reaction. The tubes were vigorously mixed for 5 min at 4°C, then centrifuged again to remove the debris. The supernatants containing chromatin fragments were recovered and diluted 10-fold with IP dilution buffer (0.01% SDS, 1.1% NP40, 1.2 mM EDTA, 16.7 mM Tris-HCl pH 8.0, 167 mM NaCl plus protease inhibitors). Antibodies were added and incubated overnight. Immunoprecipitates were collected by adding protein A/G agarose (30 µl) (Santa Cruz Biotechnology, Santa Cruz, CA) for 30 minutes at 4°C. The agarose: antibody complexes were harvested by centrifugation and washed twice for 5 min each with low salt buffer (0.1% SDS, 1%Triton X-100, 2 mM EDTA, 10 mM Tris-HCl pH 8.0, 150 mM NaCl, plus protease inhibitors), high salt buffer (same as low salt buffer but 500 mM NaCl), LiCl2 buffer (10 mM Tris-HCl pH 8.0, 250 mM LiCl, 1 mM EDTA, 1% NP40, 1% Deoxycholate plus protease inhibitors) and then TE (10 mM Tris-HCl pH 7.4, 1 mM EDTA plus protease inhibitors). The beads were resuspended in 100 µl of 1% SDS plus protease inhibitors and incubated 5 min at 65°C to elute the immunoprecipitates. The samples were centrifuged and the supernatants were transferred to new tubes, NaCl was added to a final concentration of 0.3 M and they were incubated at 65°C overnight to reverse the crosslinks. Then the samples were mixed with 2.5 volumes of cold ethanol, incubated at −20°C for 2 hr and centrifuged for 10 min at 14,000× g to collect the DNA pellet, which was rinsed with 70% ethanol, re-centrifuged, drained and resuspended in 100 µl of 10 mM Tris-HCl pH 8.0. The samples were supplemented with 1 µl Proteinase K (10 mg/ml, Sigma-Aldrich) and 1 µl RNase A (100 µg/ml, Sigma-Aldrich) and incubated at 42°C for 2 hr. The final DNA sample was purified using a Qiagen MinElute PCR Purification Kit, following the manufacturer's instructions, and samples of the final product were assayed by quantitative real-time PCR using gene-specific primer sets (Table S1). ChIP was performed with anti-Myb monoclonal 1.1 antibodies (Millipore, Billerica MA), control non-immune serum (10 µl), anti-FLAG (Sigma, St. Louis MO) or with a rabbit anti-peptide antiserum prepared by using a peptide (HQGTILDNVKNLLEFAE) from the c-Myb transcriptional activation domain as antigen (Ab 1493). In the Jurkat cell assays, these two antibodies gave very similar results, although only the data obtained with the commercial antibodies are presented here.

### Fix-Sort-ChIP Assay

The Fix-Sort-ChIP method combines fixation for chromatin immunoprecipitation and cell cycle specific flow sorting techniques, and is based on previously published methods [49], but the complete procedure is provided here for clarity. Jurkat cells (ATCC) were cultured in RPMI medium supplemented with 10% fetal bovine serum (Invitrogen, Carlsbad CA). Approximately 1×10^7^ asynchronously growing cells were fixed by adding 1% formaldehyde directly to the growth medium for 10 min. The excess formaldehyde was quenched by adding Glycine to 0.125 M for 5 min prior to harvesting the cells by centrifugation. The cells were washed in PBS then resuspended at a density of 1×10^6^ per ml in PBS containing 10 µg/ml Hoechst 33342 DNA content dye (Invitrogen, Carlsbad CA) and incubated for 30 minutes at 37°C [47]. After fixation, the cells were centrifuged and resuspended in 1 ml of growth medium containing 10 µg/ml Hoechst 33342 dye. Fixed and stained cells were sorted using a MoFlo cell sorter (Beckman Coulter, Fullerton CA). Hoechst fluorescence was excited with UV wavelengths of an argon ion laser (40 mw, 351–368 nm multiline) and detected at 390–420 nm (405/30 band pass filter). Cells were electronically gated on forward and side scatter signals excited by a second argon ion laser tuned to 488 nm to eliminate debris. An additional electronic gate was constructed about the diagonal region of a dot plot of pulse area vs pulse height for Hoechst fluorescence emission signals to eliminate off-diagonal events corresponding to doublets and higher order aggregates. Sorting was performed in single drop, purify mode to achieve maximal purity of fractions, based on DNA content. Sorted fractions were centrifuged and the cell pellets were stored at −80°C.

For chromatin immunoprecipitation, approximately 1×10^6^ cells from each sorted cell cycle fraction were resuspended in 500 µl of cell lysis buffer (20 mM Tris-HCl pH 8.0, 85 mM KCl, 0.5% NP-40 plus protease inhibitors mix: 1 mM each of chymostatin, leupeptin, antipain and Pepstatin A and 1 mM each of Phenylmethanesulfonyl fluoride and benzamidine, all from Sigma-Aldrich, St. Louis MO), incubated on ice for 10 min then lysed by passing up and down 2x through a 26 G needle. The nuclei were recovered by centrifugation for 10 min at 14,000 RPM in the microcentrifuge and resuspended in 100 µl of 50 mM Tris-HCl pH 8.0 containing 5 mM CaCl_2_ plus protease inhibitors. The chromatin was sheared by adding 6 U of micrococcal nuclease (USB, Cleveland OH) for 10 min at 37°C, followed by adding EDTA to 10 mM and SDS to 1% and by heating to 65°C for 5 min to stop the nuclease reaction. The tubes were placed on a vortex mixer for 5 min at 4°C, then centrifuged 10 min at top speed in the microcentrifuge (14,000 RPM) to remove the debris. The supernatants containing chromatin fragments were recovered and diluted 10-fold with IP dilution buffer (0.01% SDS, 1.1% NP40, 1.2 mM EDTA, 16.7 mM Tris-HCl pH 8.0, 167 mM NaCl plus protease inhibitors as described above). Antibodies were added and incubated overnight. Immunoprecipitates were collected by adding protein A/G agarose (30 µl) (Santa Cruz Biotechnology, Santa Cruz, CA) for 30 min at 4°C. The agarose: antibody complexes were harvested by centrifugation and washed 2 times for 5 min each with low salt buffer (0.1% SDS, 1%Triton X-100, 2 mM EDTA, 10 mM Tris-HCl pH 8.0, 150 mM NaCl, plus protease inhibitors), high salt buffer (same as low salt buffer but 500 mM NaCl), LiCl2 buffer (10 mM Tris-HCl pH 8.0, 250 mM LiCl, 1 mM EDTA, 1% NP40, 1% Deoxycholate plus protease inhibitors) and then TE (10 mM Tris-HCl pH 7.4, 1 mM EDTA plus protease inhibitors). The beads were resuspended in 100 µl of 1% SDS plus protease inhibitors and incubated 5 min at 65°C to elute the immunoprecipitates. The samples were centrifuged and the supernatants were transferred to new tubes, NaCl was added to a final concentration of 0.3 M and then they were incubated at 65°C overnight to reverse the cross-links. Then the samples were mixed with 2.5 volumes of cold ethanol, incubated at −20°C for 2 hr and centrifuged for 10 min in the microcentrifuge (14,000 RPM) to collect the DNA pellet, which was rinsed with 70% ethanol, re-centrifuged, drained and resuspended in 100 µl of 10 mM Tris-HCl pH 8.0. The samples were supplemented with 1 µl Proteinase K (10 mg/ml, Sigma-Aldrich) and 1 µl RNase A (100 µg/ml, Sigma-Aldrich) and incubated at 42°C for 2 hr. The final DNA sample was purified using a Qiagen MinElute PCR Purification Kit, following the manufacturer's instructions, and samples of the final product were assayed by quantitative real-time PCR using gene-specific primer sets (Table S1).

### Myb Lentivirus Vector

The cDNA encoding an N-terminal FLAG-tagged human c-Myb [3] was cloned into the unique PacI site of the pHR IRES GFP lentiviral vector (kindly provided with packaging vectors by Dr. Bruce Bunnell, Tulane University) directly downstream of the human elongation factor 1 alpha promoter. Viral particles were produced by calcium phosphate transient transfection of 293 FT cells (Invitrogen, Carlsbad CA) along with the lentiviral packaging plasmid delta 8.9 and the pMD.G plasmid expressing the vesicular stomatitis virus glycoprotein. Cell culture supernatant was collected twice in 24 hr intervals post transfection and viral supernatant was concentrated by ultrafiltration using an Ambion Ultracell 100 kDa NMWL filter unit (Millipore, Billerica MA). Jurkat cells were transduced in the presence of 8 µg/ml of polybrene (Sigma, St. Louis MO) and GFP+ cells were recovered by cell sorting.

## Supporting Information

Table S1 **Real time PCR primer pairs.** The table describes the primer pairs used for Quantitative Real Time PCR (QPCR) for the promoters listed in the first column. The last two are control primer sets.
(DOC)
