# Supplementary material for: Dramatic Repositioning of c-Myb to Different Promoters during the Cell Cycle Observed by Combining Cell Sorting with Chromatin Immunoprecipitation
Source: PLoS One. 2011 Feb 22;6(2):e17362. doi: 10.1371/journal.pone.0017362 (PMC3043100; doi:10.1371/journal.pone.0017362)
Supplement: Table S1 — Real time PCR primer pairs. The table describes the primer pairs used for Quantitative Real Time PCR (QPCR) for the promoters listed in the first column. The last two are control primer sets. (DOC) [file pone.0017362.s001.doc]

# Supplemental Information for:

# Dramatic repositioning of c-Myb to different promoters during the cell cycle observed by combining cell sorting with chromatin immunoprecipitation

Authors:

Anita M. Quintana*, Ye E. Zhou, Janeth J. Pena, John P. O’Rourke and Scott A. Ness

Table S1. Real time PCR primer pairs

| **Name** | **Forward Primer** | **Reverse Primer** | **Location*** |
| --- | --- | --- | --- |
| CCNB1 | TTGTGCCCCACCTTAAT | CATGTTGATCTTCGCCTTATT | +717 to +837 |
| CCNE1 | GGCCCCCTCCTCTTCTAGTA | CAGGTCCTCAAAGGCGTCTTA | -4524 to -4327 |
| CXCR4 | GCACCTGACCCTAGTGATGCT | TTCAGGAGGTAAAAGGAGTAAAGA | -442 to -525 |
| KIT | GCGGTGCCAGGAGCTCCTAA | CTCCCCAGACAATAAAGGTCGACT | -444 to -772 |
| GAPDH | GTGCAGGAGTTCATGACCAAC | CACCTGGGCTAGAGTAC | -245 to -425 |
| CCNE1-R | CTGGAGGTGGCTGGTGTACT | CCATCCTTCTCCACCAAAGA | N/A |
| PCR Clone | AGTTAATTAACCACTGCTTACTGGCTTATCG | AGTTAATTAACAACAGATGGCTGGCAACTA | N/A |

*Relative to transcriptional start site.
